# Intraoperative radiotherapy with electrons: fundamentals, results, and innovation

**DOI:** 10.3332/ecancer.2013.339

**Published:** 2013-08-15

**Authors:** FA Calvo, CV Sole, R Herranz, M Lopez-Bote, J Pascau, A Santos, A Muñoz-Calero, C Ferrer, JL Garcia-Sabrido

**Affiliations:** 1 Department of Oncology, Gregorio Marañón General University Hospital, Madrid 28007, Spain; 2 School of Medicine, Complutense University, Madrid, Spain; 3 Department of Radiotherapy and Oncology, Radiation Medicine Institute, Santiago, Chile; 4 Department of Radiotherapy and Oncology, Gregorio Marañón General University Hospital, Madrid 28007, Spain; 5 Department of General Surgery, Hospital Gregorio Marañón General University Hospital, Madrid 28007, Spain; 6 Radiotherapy and Oncology, Provincial Hospital of Castellon, Castellon 12002, Spain; 7 Institute of Research Investigation, Gregorio Marañón General University Hospital, Madrid 28007, Spain

**Keywords:** intraoperative radiotherapy, RADIANCE, MEDTING, disease-free survival, global survival, multidisciplinary, standardisation, innovative activity

## Abstract

**Rationale and objectives:**

To analyse the programme activity and clinical innovation and/or technology developed over a period of 17 years with regard to the introduction and the use of intraoperative radiotherapy (IORT) as a therapeutic component in a medical–surgical multidisciplinary cancer hospital.

**Material and methods:**

To standardise and record this procedure, the Radiation Oncology service has an institutional programme and protocols that must be completed by the different specialists involved. For 17 years, IORT procedures were recorded on a specific database that includes 23 variables with information recorded on institutional protocols.

As part of the development and innovation activity, two technological tools were implemented (RADIANCE and MEDTING) in line with the standardisation of this modality in clinical practice.

**Results:**

During the 17 years studied, 1,004 patients were treated through 1,036 IORT procedures. The state of the disease at the time of IORT was 77% primary and 23% recurrent. The origin and distribution of cancers were 62% gastrointestinal, 18% sarcomas, 5% pancreatic, 2% paediatric, 3% breast, 7% less common locations, and 2% others. The research and development projects have generated a patent on virtual planning (RADIANCE) and proof of concept to explore as a professional social network (MEDTING).

During 2012, there were 69 IORT procedures. There was defined treatment volume (target or target region) in all of them, and 43 were conducted by the virtual planning RADIANCE system. Eighteen have been registered on the platform MEDTING as clinical cases.

**Conclusion:**

The IORT programme, developed in a university hospital with an academic tradition, and interdisciplinary surgical oncology, is a feasible care initiative, able to generate the necessary intense clinical activity for tending to the cancer patient. Moreover, it is a competitive source for research, development, and scientific innovation.

## Introduction

**Figure 1:**
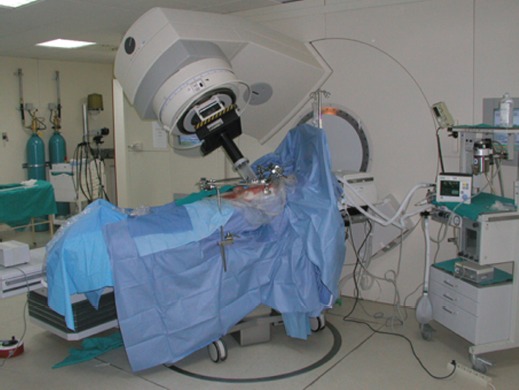
Technical description HGUGM IORT.

Intraoperative radiotherapy (IORT) is a procedure in which ionising radiation is administered during surgery. The technique involves the precise application of a high dose of radiation to the target volume area or region of interest, with minimal exposure to healthy tissue, which can be displaced and/or protected during the procedure (Figure 1) [1].

IORT is driven by a multidisciplinary approach in cancer treatment and emphasises the interaction between surgery and radiation, thus reducing the chances of a residual tumour, favouring effort at the surgical resection. This implies a minimisation of the tumour burden, maximising the radiobiological effects of a single dose and high irradiation. Its use as a component of treatment in combination with other modalities (radiotherapy, chemotherapy, maximal surgical resection) is feasible and practical if such multidisciplinary cooperation is close and coordinated. The use of IORT as a therapeutic tool is based on the realisation that tolerable doses of external radiation therapy are often insufficient to achieve control of locally advanced cancer.

European pioneers in the field of IORT are Spain, Italy, Austria, and Germany. Although it is true that most of the scientific information generated before 1980 was anecdotal and of little practical influence in the oncology community, the first known similar experiment of IORT was documented by Comas and Prio in 1905 [2], in a case of endometrial cancer. The modern approach to IORT began with studies by Abe at the University of Kyoto [2] in the 1960s by using high doses (25–30 Gy) of gamma rays from cobalt unit and betatron electrons. In the 1970s, special facilities dedicated to performing IORT procedures with conventional linear accelerators were set up at Howard University Hospital and Massachusetts General Hospital. In the early 1990s, mobile linear electron accelerators and low-energy miniature x-ray machines were introduced into clinical practice in a series of radiation therapy centres worldwide.

Regarding expansion of IORT in Europe, the number of institutions carrying out this treatment modality has been increasing in all countries. Experiments on the value of IORT are described in different tumour locations, histological types, and stages of the disease (primary or recurrent). Although some institutions in the United States were more active at the time of formalising IORT in the 1980s to mid-1990s, Europe has had an active group from the year 2000 regarding multi-institutional collaboration by grouping homogeneous cases (pooled analysis). The goal we are trying to achieve through this partnership is the standardisation of the technique in clinical practice. Therefore, and in order to promote scientific and professional approach in the activity of the IORT, the International Society of Intraoperative Radiation Therapy (ISIORT) was founded in 1998 in Pamplona, and the European section of ISIORT (ISIORT-Europe) was activated in 2006. Among the activities of ISIORT-Europe is a database that has collected information from affiliated centres to facilitate retrospective analyses, to assist in the design of prospective trials, to help standardise treatment modalities and clinical developments, and to promote technical order to satisfy a variety of needs in cancer treatment [2].

In response to the technological gap that assumes the absence of dosimetric planning in IORT treatments, specific systems are being developed for treatment planning in order to assist in the process of therapeutic decision making, to document radiosurgical techniques, and to define the volume in white-beam dosimetric distribution (GMV-RADIANCE). The pre-, intra-, and post-planning tools will help tackle clinical and technical challenges as well as being appropriate tools in the virtual training of expert groups. RADIANCE is a European research project initially based on a scientific partnership between the Gregorio Marañón University Hospital (HGUGM) and GMV [3, 4].

Finally, with the aim of exchanging information on clinical activity and achieving multidisciplinary interaction in an easier, faster, and more complete manner, the MEDTING clinical case record was implanted in the HGUGM platform. This tool allows for better communication between specialists offering a new model of collaborative oncology.

## Material and methods

### HGUGM Programme and Institutional and multidisciplinary protocols

The IORT Institutional Programme was created 18 years ago at HGUGM to adapt and implement the IORT with an electron methodology programme in a university hospital public health system, which experiences high load and complexity of care. The Oncology Radiation Department has the necessary instrumentation to articulate an IORT programme. Two radiation unit linear accelerators are used (SL-18 and Precise), emitting energies of 4, 6, 8, 10, 12, 15, and 18 MeV. Applicators tailored by the electron linear accelerator are employed, which allow the electron beam collimation with diameters of 5–15 cm in 1 cm increments with bevelled angle options of 0°, 15°, 30°, and 45°. The Oncology Radiation Department has an operating room where surgical procedures are performed on IORT candidates. The patient is transported a distance of 50 m and treated with IORT on the same table where the initial surgical resection procedure is performed. The process of clinical and therapeutic decisions in the IORT is agreed upon by radiation oncologists and radiation physicists. To this end, one must consider the preoperative CT, pre-resection surgical findings, and post-resection surgical specimen characteristics, defining the technical parameters of the IORT procedure and manoeuvres necessary to protect healthy tissues. There is a protocol that must be followed by all the different medical specialists who have treated the patient during the procedure: radiotherapy technicians, nurses, surgeons, anaesthetists, and radiation oncologists. The following protocols exist currently: radio-surgical-anaesthetics, radiation physics, radiation technicians, and nurses. This methodology has allowed the registration of clinical and therapeutical data. The development of these documents involves three periods: before, during, and after IORT [5].

### Intraoperative electron radiation therapy database: prospective therapeutic clinical information

From January 1995 to March 2012, IORT procedures performed at HGUGM have been recorded in a database that incorporates the following demographical, clinical, and technical information: (a) anonymous patient data such as age, gender, and performance status according to the Karnofsky scale; (b) tumour data including histology staging according to the TNM classification, if it is a primary tumour or a recurrence; (c) treatment data including treatment plans, surgery data, and treatment strategy; and (d) IORT specific data including the number of fields, the diameter of the applicator, and the bevel angle, energy, and type of radiation, the total dose, and the reference isodose.

### RADIANCE and MEDTING

During 2012, there were 69 procedures at HGUGM-IORT. Defined treatment volume in all of them and 43 cases (21 rectal cancers, 8 sarcomas, 6 gynaecological cancers, 5 breast cancers, 2 gastroesophageal cancers, and 1 pancreatic cancer) have been conducted following IORT planning. Of these 43 cases, 18 have been recorded on the MEDTING platform [6, 7].

## Results

From January 1995 to March 2012, data from 1,036 IORT institutional programme procedures were collected, and 1,004 patients were treated. The mean age of patients was 61 years with a range of 5 months–94 years. The gender distribution was male in 54% and female in 46% of cases. The state of the disease at the time of IORT was 77% (796) primary and 23% (240) recurrent. The intent of therapy was curative in 98% of cases and palliative in 2%. The distribution of cancers comprised 641 gastrointestinal (62%) (including 553 (53%) colorectal and 88 (9%) oesophagogastric), 190 (18%) sarcomas, 55 (5%) pancreatic, 19 (2%) paediatric, 31 (3%) breast, 77 (7%) less frequent locations, and 23 (2%) others. The following cancer sites/histologies are included in the latter two categories, in order of frequency: gynaecological (cervix, endometrium, ovary, vagina, vulva), urology (kidney, bladder, testis), chordoma, hepatobiliary, adrenal schwannoma, lymphoma, metastatic thyroid cancer sacroiliac, and spleen.

The type of surgery was categorised as radical resection in 89% of cases, microscopic residue in 3%, macroscopic residue in 7%, and no resection in 1% of cases. Rectal cancer and sarcomas, due to the volume of patients treated, are the most common tumours that received each type of surgery, while cancers of the pancreas and cervix are the most commonly treated after surgery without resection (surgical staging and exposure to beam irradiation). Surgical specialties cooperating and collaboration rate are 76% general surgeons, 12% orthopaedic surgeons, 8% gynaecologists, 2% paediatric surgeons, 1% urologists, and 1% dermatologists [8].

### Clinical results HGUGM: tumour subtypes

**Rectal cancer:**in 2001, the first results were reported at HGUGM. One hundred cases of locally advanced rectal cancer T3–T4N×M0 received a neoadjuvant chemoradiotherapy treatment regimen, radical surgery with IORT in the presacral region, followed by adjuvant chemotherapy (the latter in 52 patients). With a mean follow-up of 23 months, 3% of all had a local recurrence and 14% distant metastases. Local control at four years was 94%. Disease-free survival at four years was 75% with an overall survival of 65% in the same period [9, 10].

**Gastroesophageal cancer:**to update the results in 2010, we analysed 53 patients with locally advanced carcinoma of the oesophagus and gastroesophageal junction with a main staging T3 (77%), N1 (51%), and M0. The patients received radical surgery with IORT after neoadjuvant chemoradiotherapy in 96% of cases and adjuvant chemotherapy in 28% of cases. About 15% of the patients had local recurrences and 42% had distant metastases at a 28-month follow-up. The median overall survival was 42.8 months. We identified a significantly higher local control if treatment was superimposed with IORT [11]. In 32 patients with gastric cancer, among which 44% were located in the corpus, 28% in the cardia, and the other 28% in the antrum; the topographic pattern of progression after radiotherapy intensification with IORT in the trunk coeliac was analysed. Fifty per cent of the cases are diagnosed at a stage T3N0–N1M0. All patients received radical surgery (total or partial gastrectomy) with IORT and adjuvant chemoradiotherapy. Sixteen per cent of the patients had local recurrence at 40 months of follow-up (mainly hepatic hilum). Overall survival at five years was 54.6% [12].

**Pelvic recurrence:**from 1995 to 2011, 60 patients with pelvic recurrences of rectal cancer received extensive surgery (43% multiorgan, 28% bone, and 38% soft tissue) or conservative surgery followed by IORT on the operating table. The mean follow-up was 36 months (range 2–189). During the first, third, and fifth year, local control rates were 86%, 52%, and 44%, respectively [13].

**Extrapelvic recurrence:**from 1996 to 2010, 28 patients presented with recurrences or extrapelvic metastases (67% gynaecological, 14% urological, and 14% colorectal) were treated, combining surgery with IORT. The mean follow-up was 39 months (range 1–84). During this period, 14% of patients developed a local recurrence and 54% distant metastasis. Overall survival at two and five years was 57% and 35%, respectively [14].

**Paediatric cancer:**from 1995 to 2012, 33 paediatric cancer patients (10 neuroblastoma, 7 Ewing sarcomas, 10 sarcomas, 3 fibromatosis, 1 teratoma, 1 nephroblastoma, and 1 PNET) were treated with IORT. The data on local control at five years of follow-up were 68% in neuroblastomas, 60% in sarcomas, and 57% in Ewing sarcomas. Disease-free survival at 60 months of follow-up was 52% [15].

**Breast cancer:**From 2009 to 2011, 28 patients were treated with breast-conserving surgery followed by IORT on the operating table. Mean follow-up was 22 months (range 2–34). The mean age was 64.5 years (range 52–85). The time for complete healing of the surgical wound was less than 15 days in 92.6% of cases. There were no wound infections or seroma development, with good cosmetic results in 64.3% [16].

### Clinical results HGUGM in collaboration with other centres

**Pancreatic cancer:**in the ISIORT-Europe group ‘pooled-analysis’ study, 270 patients with pancreatic adenocarcinoma T3–T4N0–N1M0 received radical surgery in 53% of cases with IORT. Twenty-four per cent received neoadjuvant radiotherapy, 40% adjuvant radiotherapy, and the remaining 36% had no additional radiotherapy. Local control at five years was 23.3%; there was an overall survival of 19 months and a five-year survival of 17.7%. The preoperative chemoradiotherapy IORT was the most efficient strategy [17].

**Sarcoma:**in the period from 1955 to 2011, the Gregorio Marañón and Ramon y Cajal hospital treated 212 patients with soft tissue sarcoma using an IORT component. The locations were extremity (138), retroperitoneum (48), and central (26). With a mean follow-up of 3.3 years, the local recurrence-free survival, overall survival, and disease-free survival at five years were 99%, 65%, and 71%, respectively [18].

## RADIANCE: experience 2012

**Figure 2:**
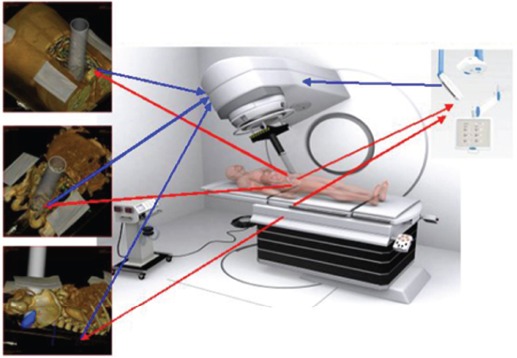
Unmanned concept, planning, and pre-robotic automation IORT treatments.

**Figure 3:**
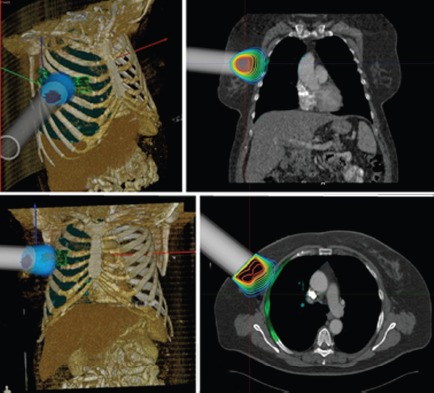
IORT simulation using RADIANCE breast cancer.

**Figure 4:**
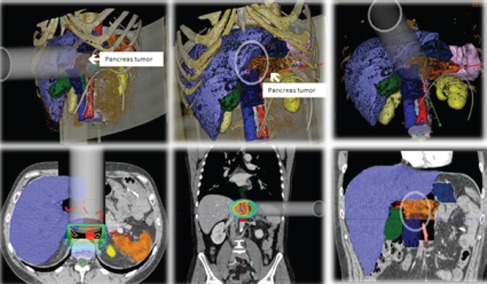
IORT simulation unresectable pancreatic adenocarcinoma with RADIANCE.

RADIANCE is the only tool designed and patented for IORT planning with US Food and Drug Administration approval and the European Community mark. Using a graphics engine, the system is capable of generating a three-dimensional image of the patient from a CT or MRI before surgery. Through segmentation on the axial, coronal, and sagittal planes, the user can generate a volumetric 3D image of organs at risk and the tumour bed. The system then determines the surgical field in real time and optimises the parameters of the applicator (cone diameter, bevel, energy, and dose). Finally, the platform allows optimisation of treatment parameters, by calculating a dose–volume histogram 4.7 (Figures 2–4).

## MEDTING

**Figure 5:**
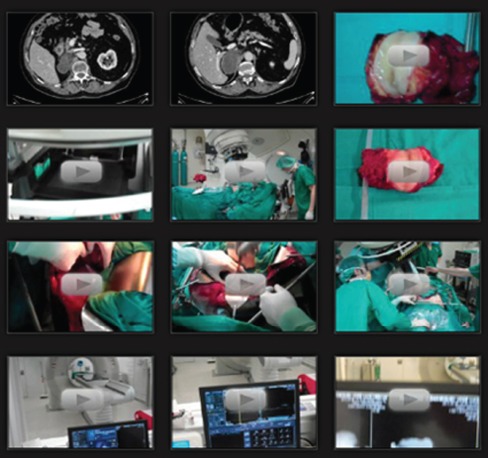
MEDTING viewer: placement of images obtained from the medical history or surgical filming.

**Figure 6:**
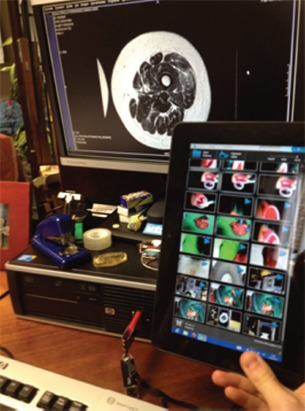
System filming and recording of images configured for automatic transfer MEDTING platform.

MEDTING digital platform is a comprehensive study focused on clinical cases. The project initially consisted of 28 doctors and 5 departments, initiated at HGUGM with the aim of exchanging information to strengthen the clinical activity and health education among professionals. In this platform, any medical professional can browse and share clinical cases, images, or videos, incorporating their own files (Figures 5and 6). The proof of concept has had the participation of 225 doctors, 120 medical students, and 39 departments in three hospitals. The impact of MEDTING lies in the opportunity it offers to different working groups to get all the information relevant to the multidisciplinary treatment decision in cancer patients and support the activities of the tumour board.

Within MEDTING, there is a specific subgroup for evaluating IORT candidate patients, enabling a more agile and dynamic process. Today we can find a record of 18 cases treated with IORT online, of which 7 are rectal tumours, 4 are sarcomas, 3 are breast, 2 are cervix, 1 is pancreatic, 1 is gastric, and 1 is endometrial cancer. Its assistance and training projection in the field of IORT are still in pilot phase (Figure 5) [8].

## Discussion

IORT has gone through three decades of development, implementation, oncology indications selection, and technological innovation (virtual planning and miniaturisation of linear accelerators for use in the operating room). Its scientific influence has been considered (684 publications registered in PubMed between 1997 and 2007 with a cumulative impact factor of more than 1300, which increases between 2007 and 2010 by 104 publications and a cumulative impact factor of 277,856). Scientifically, the most productive institutions are from Europe (46%), North America (32%), and Japan (14%). In 2012, the bibliometric analysis of articles in relation to IORT includes 858 articles originating from 281 medical institutions (32 countries) with clinical content (78%), physical and/or technology content (18%), or radiobiological content (4%). This knowledge base available in the medical literature promotes slow, but recognisable, expansion of the IORT. The most obvious limitation is the extra complexity introduced into already complex structures (teaching hospitals), and the imperative that its expansion is met with interdisciplinary oncology collaboration, which is only viable in institutions with proven ability to work in collaborative welfare projects of continuous innovation. The main objective is to achieve, through the active collaboration between different centres at the European level, the necessary studies to obtain reliable and practical results to promote and optimise the development of this treatment modality. The clinical results obtained in different studies carried out at HGUGM, along with other contributions and national and international experiments, allow us to recommend the use of IORT as a component of cancer treatment.

A specific analysis of the contribution of IORT in different human neoplastic diseases can be summarised as follows:

— Paediatric cancers: the use of external radiotherapy and IORT should be considered as an integral part of multimodality therapy for paediatric cancer to improve local control rates. External radiation therapy can be omitted in some patients while minimising its aftermath [15, 19].
— Soft tissue sarcomas: the use of IORT combined with external radiotherapy in the treatment of soft tissue sarcomas offers high local control, allowing functional limb preservation. The identification of peripheral nerves in the radiation field is IORT dose limiting the risk of symptomatic neuropathy [18].
— Oesophageal and gastroesophageal cancer: applying overlay on the circumferential margin of the gastro-oesophagectomy allows better control of the disease although has little impact on overall survival. The use of IORT on stomach cancers is feasible in combination with basic therapy in the treatment of stomach cancer and achieves good locoregional control [11, 12].
— Pancreatic cancer: local control and survival were compared between different therapeutic strategies (pre-radio + IORT, IORT + post-radio, IORT). The results showed that the application of preoperative external radiotherapy improved the favourable effects of IORT in terms of local control and survival [17].
— Rectal cancer: use of an IORT overlay component applied over the region of residual disease or those at high risk of postoperative relapse (usually posterior or presacral space hemipelvis); together with other radical treatment strategies of locally advanced rectal cancer, it contributes to the improvement of local control rates [9, 10].
— Breast cancer: a growing interest in using IORT for early-stage breast cancers, since the use of this technique can reduce the amount of irradiation (partial breast radiotherapy) and clinical effects on structures such as skin, lung, or heart. It is a model that has been successfully explored through the use of a massive single dose (21 Gy), avoiding the need for external radiation. Despite these advantages, further studies are necessary in order to make a correct assessment of the role of IORT in new clinical situations (later stages) [19].

Given that the therapeutic approach in the fight against cancer is based on interdisciplinary communication, improving this requirement will optimise the diagnostic and therapeutic approach for cancer patients. In this scenario, the MEDTING platform enables full and simple communication. MEDTING allows the sharing of all clinical and laboratory data, imaging tests (CT, MRI, Rx), tumour histological images, and the associated pathological data, thus providing the continuous and real-time data that are needed to carry out the diagnostic and therapeutic strategy in a consensual manner, also allowing other health professionals, including other hospitals, contribute their experience and opinion. The standardisation of clinical practice in the treatment modalities of super-specialised multidisciplinary institutions with complex care processes, such as the IORT technique, is a valuable support for ensuring quality of care. The sub-IORT platform contributes to this normalisation with the added value of thorough clinical evaluation on a case-by-case basis [7].

The development of effective tools for IORT planning has been limited due to the complexity of reproducing the surgical procedure and anatomical resection and the lack of standardisation in the virtualisation of these processes. Establishing and consolidating an IORT programme involves many aspects from an institutional point of view, since it requires structural organisation and human resources, such as modified accelerators; a multidisciplinary team; and coordinated action of surgeons, anaesthetists, medical physicists, radiation oncologists, and nurses. The IORT planning system RADIANCE compares various therapeutic possibilities correlating with the clinical case, which facilitates the optimal selection of the parameters and early implementation of IORT. This feature is a new, previously unavailable contribution. A study that demonstrates the system’s ability to meet the needs of users in different clinical scenarios now exists. The current developmental stability has allowed the installation and evaluation of RADIANCE in four hospitals in Spain (HGUGM, Clínica La Luz, HPC, Ramón y Cajal Hospital). These centres are collaborating on the development of new protocols for IORT, involving simulation, planning, and tele-guided automation (pre-robotics) using this tool [4, 6, 20].

## Conclusion

IORT has been implemented and is available in a wide number of international hospitals and academic institutions and offers an attractive interdisciplinary oncology initiative that has been proven capable of generating intense activity-directed clinical oncology patient care and is a competitive source of research, development, and scientific innovation. One can venture that IORT, as a superselective radiotherapy overprint component, will expand and modify its indications in clinical and therapeutic models that benefit from resective surgery and localised radiotherapy, which in turn are needed to promote practically non-toxic local tumour control [1].

## Acknowledgement

This work was supported in part by grants from the Spanish Minister of Science and Innovation (Projects FIS PI 11 02908, Precise and Innpacto).
